# Axon Guidance Mechanisms for Establishment of Callosal Connections

**DOI:** 10.1155/2013/149060

**Published:** 2013-02-24

**Authors:** Mitsuaki Nishikimi, Koji Oishi, Kazunori Nakajima

**Affiliations:** Department of Anatomy, School of Medicine, Keio University, 35 Shinanomachi, Shinjuku-ku, Tokyo 160-8582, Japan

## Abstract

Numerous studies have investigated the formation of interhemispheric connections which are involved in high-ordered functions of the cerebral cortex in eutherian animals, including humans. The development of callosal axons, which transfer and integrate information between the right/left hemispheres and represent the most prominent commissural system, must be strictly regulated. From the beginning of their growth, until reaching their targets in the contralateral cortex, the callosal axons are guided mainly by two environmental cues: (1) the midline structures and (2) neighboring? axons. Recent studies have shown the importance of axona guidance by such cues and the underlying molecular mechanisms. In this paper, we review these guidance mechanisms during the development of the callosal neurons. Midline populations express and secrete guidance molecules, and “pioneer” axons as well as interactions between the medial and lateral axons are also involved in the axon pathfinding of the callosal neurons. Finally, we describe callosal dysgenesis in humans and mice, that results from a disruption of these navigational mechanisms.

## 1. Introduction

 Interhemispheric connections are essential components of the complex neural network in eutherian animals [1, 2]. Among such connections, the corpus callosum (CC) is the most prominent commissural connection, composed of callosal axons, in the brain. In humans, the corpus callosum consists of about 200 million axons, making it the most prominent fiber tract within the central nervous system [3, 4]. Many studies have clarified the molecular mechanism involved in the development of the CC in humans using mouse experiments [5]. 

 Callosal neurons are mostly found in layers II/III and layer V of the cerebral cortex in rodents [6]. Recently, molecules related to the identities of the general or subtypes of cortical neurons have been disclosed. Alcamo et al. reported that Satb2, a DNA-binding protein, has a key role in the specification of callosal neurons and the formation of corticocortical connections [7].

 Developmentally, callosal axons from layer V first start to project to the contralateral targets, and callosal axons from the upper layers follow the preexisting axons. After the callosal axons start to elongate, they are guided by many cues within their pathfinding route [6]. Although the importance of such cues in the development of callosal axons has been known for over 30 years [8], it still remained unclear until recently how these cues help callosal axons encountering them to project precisely to their targets. Recent studies have, however, revealed the detailed mechanisms in the regulation of axon guidance by these structures. Midline structures, which consist of glia and neurons, express or secrete short- or long-range guidance molecules [9]. In the contralateral cortex, where the callosal axons terminate, interactions with postsynaptic neurons play important roles, in an activity-dependent manner, in ensuring proper projections [10–12].

 In this paper, we first focus on how the callosal axons are guided by the cues that they encounter, namely, the (1) midline structures and (2) neighboring axons, from the time that they start to grow until they reach their targets in the contralateral cortex. Then, we describe the activity-dependent development of the interhemispheric connections. Finally, the consequences of callosal agenesis in humans and mice are reviewed.

## 2. Callosal Axon Guidance by the Midline Structures during Development of the CC

**Figure 1:**
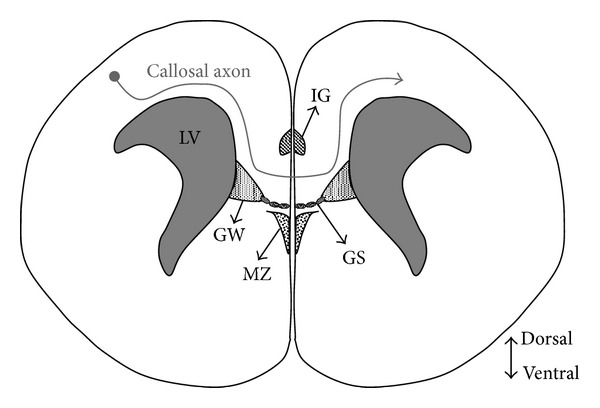
Glial populations around the midline. The locations of the midline glial populations on a coronal section of the E15.0 mouse brain are shown. These populations are mainly composed of four structures: the glial wedge (GW), the indusium griseum (IG), the midline zipper (MZ), and the glial sling (GS). These populations guide the growth of the callosal axons and help them cross the midline in the CC. LV: lateral ventricle.

The midline structures mainly consist of glial populations, but also contain neuronal populations [13]. The role of the midline glial structures in the formation of the CC was first reported by Silver et al. [8]. In the mouse brain, these glial structures have been shown to already exist on embryonic day (E) 15.0 and can guide the growth of callosal axons [8, 14, 15]. The midline glial structures mainly consist of four structures: the glial wedge (GW), the indusium griseum (IG), the midline zipper (MZ), and the glial sling (GS) [8, 16] (Figure 1). The MZ is thought to be required for the fusion of the two hemispheres, which facilitates the passage of axons across the midline [9, 17]. The other structures are responsible for promoting the crossing of at least the callosal axons [18–23]. These structures help the callosal axons find their correct path by secreting or expressing guidance molecules. Interestingly, Shu and Richards have illustrated that correct orientation as well as the presence of the GW is required for callosal axons to turn toward the midline; in one experiment, when the GW was replaced with 180° rotation (medial to lateral), the axons turned away from the midline [15]. 

**Figure 2:**
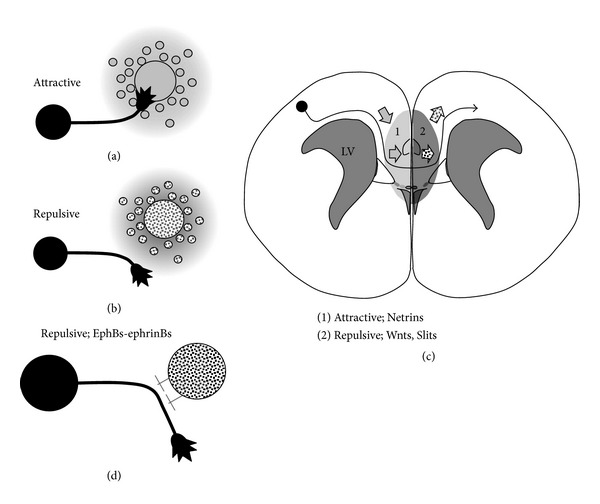
Glia-axon interactions in the development of callosal axons. (a) and (B) Axon guidance by long-range molecules, attractive (a) or repulsive (b) signals. Glial populations (gray and dotted circles in (a) and (b), resp.) secrete guidance molecules, forming a concentration gradient, which navigates the callosal axons during their development (c). (d) Axon guidance by short-range molecules. Repulsive molecules expressed on the cell membranes navigate callosal axons through repulsive and bidirectional cell-cell contact functions.

 Studies have gradually uncovered the molecules secreted by the midline structures for callosal axon guidance. The axon guidance cues for callosal neurons secreted by the midline structures have been classified into two types: long range (Figures 2(a)–2(c)) and short range (Figure 2(d)). The long-range guidance molecules are secreted by the midline glial populations, forming a concentration gradient and helping callosal axons pass through the CC with attractive (Figure 2(a)) and repulsive signals (Figures 2(b) and 2(c)). Slits [15, 24, 25], Wnts [26], Netrins [27, 28], Draxin [29], and Semaphorins [30] are some of the reported long-range guidance molecules. Recent studies have also clarified new roles for some of these guidance molecules. For example, Unni et al. have suggested a novel role of Slits in regulating the positioning and maturation of the midline glial populations, presumably independent of the activity of its receptor, Robo1, in addition to its role as a repulsive axon guidance cue [31]. Wnt5a not only promotes axon outgrowth as a long-range guidance molecule, but also serves as a short-range repulsive axon guidance cue [32, 33]. 

In addition to the midline structures, other cell populations have also recently been shown to play roles in the formation of the CC. GABAergic and glutamatergic neurons that transiently exist within the CC have been shown to be able to attract callosal axons [34]. The meninges have also been reported to be involved in the development of the CC. BMP7 secreted by the meninges has been shown to inhibit the outgrowth of callosal axons, potentially preventing early formation of the CC [35].

 The short-range molecules guide axons through transmembrane or membrane-associated proteins (Figure 2(d)). The ephrin/Eph signaling system is one of the best-known examples. Eph receptors are divided into two subclasses, A and B, according to the sequence homology and binding affinity for their ligands, ephrins A and B, respectively [36, 37]. Although the ephrin/Eph system signals through Eph tyrosine kinase receptors, ephrins can also transduce reverse signals into the cell in which they are expressed [38]. The EphB receptors and ephrin B ligands have been well studied and shown to play important roles in callosal axon pathfinding [39, 40]. Importantly, the complementary expression of multiple ephrin B ligands and EphB receptors in the callosal axons and midline structures has led to the hypothesis that interactions occur between the Eph receptors in callosal axons and ephrins in the midline structures or vice versa, although it is also possible that the interactions occur between callosal axonal fibers [40]. The expression of ephrin B ligands in the callosal axons is suggestive of the involvement of reverse signaling, and Bush and Soriano showed that ephrin-B1 reverse signaling is critical for callosal axon pathfinding, which requires the binding of the PSD-95/Dlg/ZO-1 (PDZ) domain-containing proteins for the transduction of this reverse signal [37]. 

## 3. Callosal Axon Guidance by Other Axons during Development of the CC

**Figure 3:**
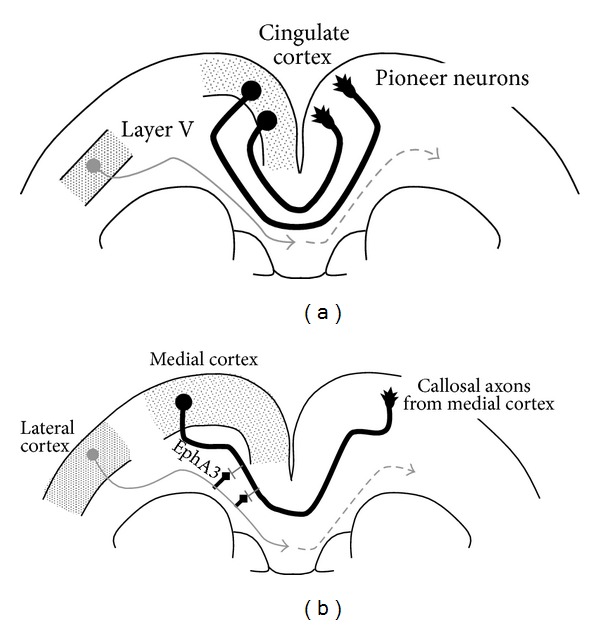
Axon-axon interactions in the development of callosal axons. (a) Navigation of callosal axons by “pioneer” neurons. Pioneer neurons, which are located in the region extending from the presumptive cingulate cortex to the hippocampus, first extend their axons to form the path of the commissural axons. Then, on E17.0, the most early “follower” axons originating from layer V follow the pioneer neurons. (b) Interaction between the medial and lateral cortex-derived callosal axons through EphA3. The axons from the medial (roughly corresponding to the cingulate, motor and medial part of the primary somatosensory cortices) and lateral cortices (roughly corresponding to the areas around the secondary somatosensory cortex) pass through the dorsal and ventral half of the CC, respectively. Repulsive effects between the medial and lateral cortical axons contribute to their correct pathfinding in the CC. EphA3 expressed on the lateral axons mediates, at least in part, this interaction between the medial and lateral axons.

 “Pioneer” neurons represent one of the most important players in callosal axon guidance by other preexisting axons during CC development [41]. On E15.5 in mice, the axons of the pioneer neurons, which originate from the cingulate cortex, cross the midline and enter the contralateral cortex (Figure 3(a)) [42, 43]. It has been shown that CC genesis is triggered by these pioneer axons [39, 42–45]; pioneer axons are the first to form the path for the commissural neurons through interactions with several cues, including the midline structures, and on E17.0, the most early “follower” axons from layer V follow those of these pioneer neurons [42, 43, 46] (Figure 3(a)). An accepted view is that the “follower” axons utilize their direct interactions with the “pioneer” axons to find their correct path of growth, although the molecular mechanism of such interaction remains unclear. Interestingly, Piper et al. described the molecular mechanisms driving the guidance of the cortical pioneers during development. They demonstrated that Neuropilin 1 expressed on the cingulate pioneers plays a crucial role in the crossing of the midline by the “pioneer” axons through interaction with multiple class 3 semaphorins expressed around the developing CC [45].

 While many studies have revealed the indispensable roles of the interactions between the callosal axons and the midline structures, it is still unclear whether axon-axon interactions play important roles in callosal axon pathfinding. Although increasing evidence has revealed the importance of these interactions in other systems, such as the retinal, spinal and olfactory systems [47–50], the involvement of such axon-axon interactions in CC development remains to be explored in detail. Nishikimi et al. have recently reported repulsive interactions between callosal axons originating from the medial and lateral cerebral cortices (Figure 3(b)). Based on a previous study by the same group [51], they focused on EphA3, which is preferentially expressed in the callosal axons from the lateral cerebral cortex, and found that knockdown of EphA3 in the lateral cortical axons resulted in their disorganized segregation in the CC and disrupted axon pathfinding. They have suggested that EphA3 mediates, at least in part, the repulsive interactions between the medial and lateral cortical axons [52].

 So far, several studies using knockout and transgenic mice have identified molecules involved in the development of the CC [9]. However, as knockout and transgenic mice show influences of all developmental stages, analyses of these mutant mice are not necessarily sufficient for describing the primary causes of the abnormal phenotypes. Recent studies using *in utero* electroporation [53] and various culture experiments, including the stripe assay [37, 54, 55], have enabled reasonably easy analysis of each specific stage of CC development. Further experiments focusing on each step of development will be essential to understand the entire process of formation of the CC.

## 4. Activity-Dependent Development of the Interhemispheric Connections

To eventually establish interhemispheric connections through the CC, reshaping of the axons is also crucial. The callosal connections are initially exuberant and brushed up by the selective death of neurons and withdrawal and degeneration of axonal collaterals [56]. Since callosal axons start to establish synapses with specific postsynaptic neurons after entering the contralateral hemisphere [39, 57], the involvement of synaptic activity-dependent mechanisms (as well as nonsynaptic activities) in this process of reshaping of the axons has been shown by many studies [57, 58]. In the visual system, for example, the stimuli from the eyes contribute to the formation of the precise patterns of callosal axonal connections [59, 60]. 

 Importantly, although the callosal axons are generally believed to have a simple mirror projection across the CC in the contralateral hemisphere, there are also heterotopic callosal projections. In addition, the tangential distribution of the callosal axon projections is not even in the adult cortex. For example, the callosal connections are highly focused at the level of the primary areas. While these might possibly be established during the later development of the callosal axon projections (i.e., refinement and elimination), establishment of such uneven projections in the early phase of development cannot also be ruled out.

Recently, synaptic and non-synaptic activities have also been reported to be involved in the regulation of different aspects of development of the callosal projections besides reshaping of the axons [11]. Blockade of the spontaneous electrical activity of the callosal neurons resulted in abnormal projections in the somatosensory cortex [11] and visual cortex [12]. Interestingly, blockade of the spontaneous electrical activity of projection neurons such as the motor and olfactory neurons also influenced a variety of guidance and adhesion molecules that are critical for their development [61–63], suggesting that spontaneous electrical activity of the axons may also have some role in axon guidance. 

## 5. Callosal Dysgenesis in Mice and Humans

 As described above, a number of different control mechanisms are involved in the development of the interhemispheric connections, and disruption of any of these mechanisms may cause malformations of the CC. Some examples are knockout mice lacking some of the molecules involved in the formation of the midline glial structures [19–23], GABAergic neurons [34, 64, 65] or pioneer neurons [45], or the axon guidance mechanism [66]. Phenotypes of such knockout mice are quite varied and range from hypoplasia or partial dysgenesis of the CC to complete dysgenesis and formation of Probst's bundles [40, 67], which are also observed in partial dysgenesis.

 A comparison between mice and humans revealed many similarities in the development of the CC between the mouse brain and human brain [5]. Not only are the midline glial structures conserved in humans [68], but also the expression profiles of the molecules known to be involved in the formation of the CC are similar between human and mouse brains [9, 69, 70]. 

 In humans, several psychiatric, neurologic, and metabolic disorders have been shown to be associated with congenital agenesis of the CC or the surgical procedure, callosotomy [5, 71, 72]. Among the famous of these reports is the story of the patient with callosotomy who could not verbally describe the stimulation presented to his freshly disconnected right hemisphere. In subjects with complete dysgenesis of the corpus callosum, many items of neuropsychological evaluation are at the lower end of the normal range [72]. Paul et al. described that despite having normal IQ, individuals with complete dysgenesis of the CC show impaired social intelligence, analyzing their responses to pictures from the Thematic Apperception Test [73]. Moreover, many studies have reported that major mental disorders, such as autism, attention deficit hyperactivity disorder (ADHD), and schizophrenia, may be related to the morphology of the CC [74–76]. However, the precise nature of these associations remains unclear. How could malformations of the CC have any relation to these disorders? Do the genes associated with these disorders play a role in normal CC development? Future studies on the development of the CC may help elucidate the precise nature of these associations.

## 6. Conclusion

 By integrating information between the right/left hemispheres, interhemispheric connections enable us to accomplish higher brain functions. Development of interhemispheric connections such as the CC is guided by molecules in the axonal environment, under the regulation of a number of different control mechanisms. Midline glial and neuronal populations express and secrete guidance molecules, and “pioneer” axons help in the axon pathfinding of the callosal neurons. Disruption of these navigational mechanisms may cause dysgenesis of the corpus callosum. It would be of great interest to conduct detailed investigation of the mechanisms underlying CC development, especially in view of their relevance in the pathogenesis of human disorders.
